# Surgical Classification for Preclinical Rat Femoral Bone Defect Model: Standardization Based on Systematic Review, Anatomical Analysis and Virtual Surgery

**DOI:** 10.3390/bioengineering9090476

**Published:** 2022-09-15

**Authors:** Yu Sun, Heike Helmholz, Regine Willumeit-Römer

**Affiliations:** 1 Institute of Metallic Biomaterials, Helmholtz-Zentrum Hereon, 21502 Geesthacht, Germany; 2 Department of Orthopaedics, First Hospital of China Medical University, Shenyang 110001, China

**Keywords:** rat, femur, bone defect, animal model, systematic review, virtual surgery, classification system

## Abstract

Though surgical techniques profoundly influence in vivo experiments, significant heterogeneity exists in current surgeries for inducing rat femoral bone defects. Such variations reduce the reproducibility and comparability of preclinical studies, and are detrimental to clinical translation. The purposes of this study were: (1) to conduct a systematic review of rat femoral defect models, summarizing and analyzing the surgical techniques; (2) to analyze surgical design and potential pitfalls via 3D anatomy and virtual surgeries for fostering future precision research; and (3) to establish a surgical classification system, for improving the reproducibility and comparability among studies, avoiding unnecessary repetitive experiments. The online database PubMed was searched to identify studies from January 2000 to June 2022 using keywords, including rat, femur, bone defect. Eligible publications were included for a review of surgical methods. Anatomical analysis and virtual surgeries were conducted based on micro-CT reconstruction of the rat femur for further investigation and establishment of a classification system. A total of 545 publications were included, revealing marked heterogeneity in surgical methods. Four major surgical designs were reported for inducing defects from the proximal to distal femur: bone tunnel, cortical window, segmental defect, and wedge-shaped defect. Anatomical analysis revealed potential pitfalls hindering efficient clinical translation. A classification system was established according to the anatomical region, surgical design, and fixation devices. This systematic review in combination with 3D analysis and virtual surgery provides a general overview of current surgical approaches to inducing femoral defects in rats, and establishes a surgical classification facilitating preclinical research of quality and translational value.

## 1. Introduction

Bone defect in the lower extremities is a common clinical situation resulting from trauma, infection, revision arthroplasty, tumor resection, or other disorders [1]. Orthopedic surgeons routinely use bone grafts to re-establish skeletal integrity and avoid tragic amputation surgeries, and bone grafting has become the second most common transplant procedure in the world after blood transfusion [1,2]. However, the current widely used auto- and allografts have the disadvantages of a limited supply, high postoperative complications, and risk of disease transmission [3]. Therefore, the development of safe artificial graft materials with a stable supply and bone-promoting activity has been a research priority in line with the clinical needs of replacing autologous or allogenic transplants.

As current in vitro experiments are still unable to mimic the complex and sequential in vivo bone regeneration process, animal experiments remain essential for preclinical assessment of novel biomaterials [4,5]. Historically, large animals were preferred for experiments, considering the application of surgical instruments with the same dimensions for human surgeries and thus better simulating the disease treatment process [6]. In recent years, with the development of fine surgical instruments and the application of multimodal high-resolution imaging techniques, the translational potential of the bone defect model in the rat femur has been markedly enhanced, facilitating the development of biomaterials [7,8,9,10]. Moreover, rat femoral defect models allow the introduction of complex comorbidities such as osteoporosis, diabetes, and infection at weight-bearing sites, providing superior simulation of clinical situations than calvarial defect models [11,12,13].

However, though it is well recognized that surgical techniques can profoundly influence study results, significant heterogeneity exists in current surgical methods for inducing femoral defects, and such variations undoubtedly reduce the reproducibility of experiments and are detrimental to clinical translation [14,15,16]. The relatively low translational potential and the corresponding large number of lab animals sacrificed have raised social and ethical concerns [17,18]. This pressure prompted investigators to carefully consider how to enhance the translational value of preclinical studies during experimental planning.

One feasible approach is the application of software tools to conduct virtual surgery by simulating operative procedures on skeletal models reconstructed from radiological data [19,20]. This allows precise simulation of operative treatment in a time-saving and cost-effective manner, supporting direct visualization of the surgical results, such as bone tunnel drilling and osteotomy [21]. In clinical practice, virtual surgeries have been adopted for preoperative planning of orthognathic and limb operations. For preclinical research, virtual surgery based on micro-CT reconstruction was developed to simulate hepatectomy in rats to improve the translational prospects [22,23,24].

To promote the clinical translation of novel biomaterials and musculoskeletal research, the authors conducted this evidence-based study, aiming: (1) to conduct a systematic review of the rat femoral defect model, summarizing and analyzing the surgery-related details; (2) to analyze surgical designs and potential pitfalls via 3D anatomical analysis and virtual surgeries in order to foster future precision preclinical research; and (3) to establish a surgical classification system to improve the reproducibility and comparability among studies and avoid unnecessary repetitive experiments.

## 2. Materials and Methods

### 2.1. Literature Search Strategy, Criteria, and Study Selection

**Figure 1:**
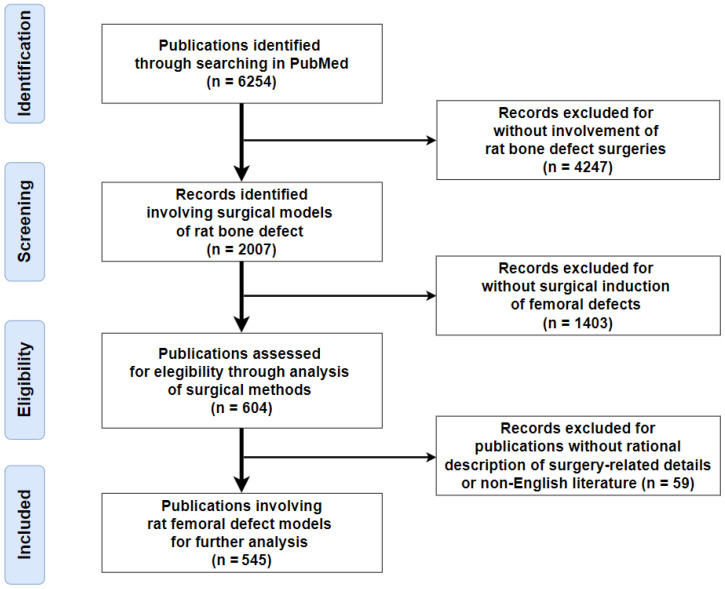
Flow diagram of the literature search and screening.

The online database PubMed was first searched for systematic reviews focusing on the surgical techniques of rat femoral defects, with no specific publications identified. Then, the authors searched for in vivo studies published in the English language from January 2000 to June 2022 involving bone defects in the rat femur using the following keyword combination: rat, femur, bone defect. The workflow used for literature retrieval, screening, and selection is shown in Figure 1. No restrictions were set regarding comorbidities such as osteoporosis or diabetes. The literature search followed the Preferred Reporting Items for Systematic Reviews and Meta-Analyses guidelines [25] and was registered in the Center for Open Science (osf-registrations-n74ve).

The inclusion criteria were: (1) in vivo studies involving surgical procedures inducing femoral defects in rats; and (2) reporting of intra-operative details for the preparation of bone defects, with radiological or histological evidence or textual descriptions supporting reproduction of the surgeries. The exclusion criteria were: (1) reviews, commentaries, or pure abstracts; (2) in vitro tests without in vivo experiments; and (3) in vivo studies using bone defects in other animal species or at other anatomical sites in rats instead of femur. The authors first browsed the titles and abstracts within the search results to screen and enroll publications based on the above criteria. Enrolled papers were further carefully checked for data extraction of the following general and surgery-related details: publication year, rat strain, gender, age, body weight, follow-up period, anatomical position of bone defect, modeling techniques (surgical procedures), and fixation devices. The preliminary search first identified 6254 publications potentially related to rat bone defects. After excluding non-relevant studies without induction of femoral defects in rats through abstracts or full texts, 545 publications were included for further analysis in this systematic review (Figure 1 and Table S1 in the Supplementary Materials).

### 2.2. Micro-CT Data and Image Processing

In order to visualize and analyze the surgical approaches used in the included studies, the authors performed 3D reconstruction of the rat femur using micro-CT data. The radiological data was obtained with whole femur samples from a previous study involving Sprague-Dawley (SD) rats. The experimental protocols were applied via local authority (Ministry for Energy Transition, Agriculture, Environment, Nature and Digitalization, Schleswig-Holstein, Germany, application number: V242-30912/2020 and V242-6462/2021) [10]. An ex vivo scan of the bone samples was performed using a VivaCT 80 scanner (Scanco Medical AG, Brüttisellen, Switzerland) at a voltage of 70 kVp, with a beam current of 114 μA and isotropic voxel size of 39 μm. For anatomical analysis and 3D rendering of virtual surgery, the image data was obtained from a male SD rat with a body weight of 400 g. To render the difference in the bone size between genders, the image data was obtained from another two SD rats of close age (male aged 20 weeks with a body weight of 560 g, female aged 24 weeks with a body weight of 338 g).

Data files from the micro-CT scanner were processed using Fiji (https://imagej.net/software/fiji/, accessed on 25 August 2022) with the BoneJ plugin and 3D slicer (http://www.slicer.org, accessed on 25 August 2022) to obtain 2D slice measurements for bone size comparison and STL files for 3D processing [26,27,28]. Anatomical analysis and virtual surgeries (simulating bone drilling, osteotomy, and bone fixation) were conducted using the Boolean Difference function in Autodesk MeshMixer (https://www.meshmixer.com, accessed on 25 August 2022) for the preparation of bone defects and establishment of the surgical classification system.

## 3. Results

### 3.1. Systematic Review of the Literature Search Results

**Figure 2:**
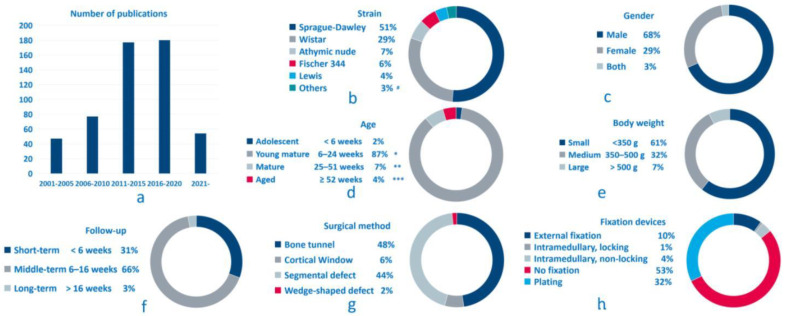
Summarized information of the included studies. (**a**) Number of publications; (**b**) rat strains involved; (**c**) gender; (**d**) age; (**e**) body weight; (**f**) longest follow-up; (**g**) modeling method; (**h**) fixation devices. Note: * including 6 weeks; ** including 6 months; *** including 12 months; **#** other rat strains: Long-Evans, Brown Norway, Dark Agouti, BB/OK, Zucker diabetic fatty; spontaneously hypertensive.

The included 545 studies were published from 2001 to 2022, with Sprague-Dawley and Wistar rats being the most frequently selected strains, accounting for 51% and 29% of all studies, respectively (Figure 2). Gender bias was detected, with a predominate focus on male rats (68%). To facilitate summarization and comparison, the authors pooled the data by stratifying the animal information by age, body weight, and longest follow-up period. The result showed that young mature rats were predominantly used (87%), and 61% of the studies selected small rats with a body weight of less than 350 g. Regarding the setting of the post-operative follow-up period, 66% were categorized as middle-term (6-16 weeks) research and only 3% as long-term research (>16 weeks).

Among all included studies, bone defects were induced at various anatomical sites, involving the whole length of the femur, from the proximal femoral neck to the distal condyle. According to the descriptions of the surgical procedure, the authors summarized four major types of surgical methods used to induce femoral defects: (1) bone tunnel: cylindrical defect prepared by drilling, transversely involving either single or two cortical layers, or longitudinally along the femoral axis into the intramedullary cavity [29,30,31,32,33]; (2) cortical window: rectangular or rounded-rectangular defect involving one single cortex [34,35,36]; (3) segmental defect: complete segmental bone resection with parallel osteotomy [37,38,39,40,41,42,43,44]; and (4) wedge-shaped defect: removal of the bone block via opening wedge osteotomy [45,46,47].

Fixations were routinely applied for segmental and wedge-shaped defects, and the studies could be categorized into five major types according to the fixation status: (1) intramedullary internal fixation, without a locking mechanism [38,39]; (2) intramedullary internal fixation, with locking pins [40,48,49,50,51]; (3) plating with screws or wires for internal fixation [52,53,54,55,56,57,58]; (4) external fixation [37,43,44,59]; and (5) no fixation [29,30,31,32,33].

Bone tunnel defects without fixation were the most frequently used surgical method (48%), followed by segmental defects with all types of fixation devices involved (44%). Cortical window and wedge-shaped defects were relatively less selected, accounting for only 6% and 2%, respectively. As to bone fixation, plating was most frequently used (32%), followed by external fixation (10%). Intramedullary nail with a locking mechanism, though with high clinical relevance, was only adopted by 1% of all included studies [40,48,49,50,51].

### 3.2. Establishment of the Surgical Classification System with Exemplar Illustrations

#### 3.2.1. Identification of Anatomical Landmarks with Micro-CT Reconstruction

**Figure 3:**
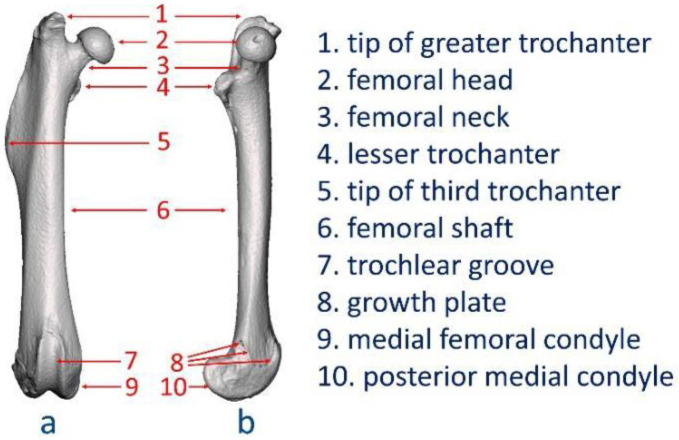
Anatomical surface landmarks: (**a**) Ventral-dorsal view; (**b**) medial view.

Most enrolled studies described the anatomical locations using less precise terms such as “proximal femur”, “distal condyle”, or “diaphysis”. The exact surgical site needed to be determined by referring to the imaging or histology images in the Methods or Results sections. To avoid ambiguities in anatomical locations, which may lead to reduced reproducibility, the authors conducted a 3D reconstruction of the femur from a male SD rat (400 g) and then defined major surface landmarks in the ventral-dorsal and medial views (Figure 3). A precise anatomical definition would not only support establishment of the surgical classification system but also facilitate the standardization of surgical practice in preclinical research.

#### 3.2.2. Definition of Anatomical Locations

**Figure 4:**
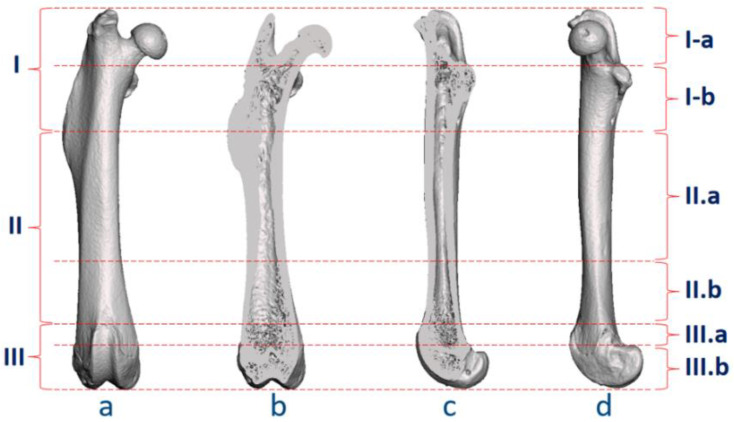
Anatomical regions in the surgical classification. (**a**) Ventral-dorsal view; (**b**) frontal plane; (**c**) sagittal plane; (**d**) medial view. Note: I: proximal femur, including I.a (femoral head and neck) and I.b (above the tip of the third trochanter); II: middle femur, including II.a (one-third of the femoral length) and II.b (between II.a and III.a); III: distal femur, including III.a (from the top of the trochlear groove to the top of the posterior condyles) and III.b (below the top of the posterior condyles).

After defining the surface landmarks, the authors established a classification for anatomical regions (Figure 4). The femur was divided into Region I (proximal femur), Region II (middle femur), and Region III (distal femur), using the tip of the third trochanter and the top of the trochlear groove as markers for boundaries. Next, Region I was subdivided into Region I.a and I.b, bounded by the proximal end of the lesser trochanter and its junction site with the femoral neck. Region II was subdivided into Region II.a and II.b, with the proximal Region II.a occupying one-third of the total femoral length, and II.b as the distal part of Region II. Region III was also divided into two parts, III.a and III.b, using the proximal top of the posterior condyle to define the boundary line.

The above regions were also characterized by different anatomical and tissue components. Region I.a comprised the femoral head and neck. Surgical exposure in this area could be difficult due to the adjacent strong muscles, tendons, and joint capsule. Only few studies involved this area, preparing bone tunnels within the femoral neck canal or greater trochanter [60,61,62,63]. Region I.b could be viewed as the intertrochanteric area between the lesser and third trochanters. The narrow cortical surface surrounding the medullary cavity was also not conducive to surgical operation. Region II.a was the main surgical site for cortical windows and segmental defects, with cortical bone as the main tissue component. Starting from Region II.b, the gradual expansion of the femoral size facilitated drilling operations, and II.b, III.a, and III.b were commonly selected in bone tunnel defect models [30,31,32,33]. As shown in the micro-CT reconstructions, Region II.b contained more cancellous bone than Region II.a. For surgeries at the distal femur, it was noted that surgical procedures in Region III.a could lead to growth plate injuries while operations in Region III.b could cause penetration of the femoral condyles or even fractures (Figure 4).

#### 3.2.3. Classification of Modeling Methods and Fixation Devices

**Figure 5:**
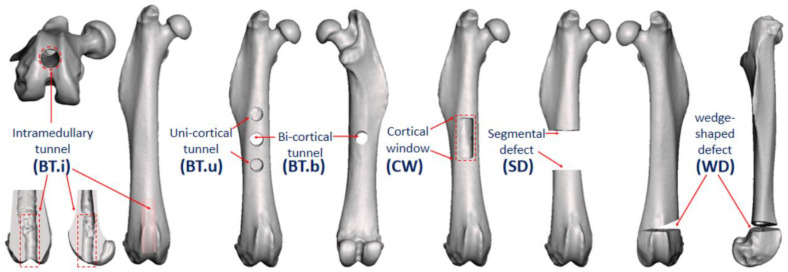
Classification of modeling methods. Note: **BT.i:** bone tunnel, intramedullary; **BT.u**: bone tunnel, uni-cortical; **BT.b**: bone tunnel, bi-cortical; **CW**: cortical window; **SD**: segmental defect; **WD**: wedge-shaped defect.

**Figure 6:**
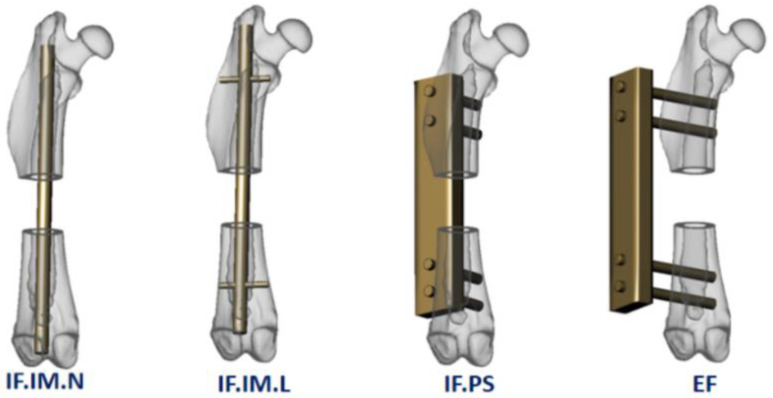
Classification of fixation devices. Note: **IF.IM.N**: internal fixation, intramedullary, non-locking; **IF.IM.L**: internal fixation, intra-medullary, locking; **IF.PS**: internal fixation, plate-screw; **EF**: external fixation.

Based on micro-CT data, the authors performed virtual surgeries to realize 3D representation of the classification of surgical methods and fixation devices (Figure 5 and Figure 6). For surgical methods, the authors adopted the framework, including four categories, as mentioned in Figure 2: bone tunnel (BT); cortical window (CW); segmental defect (SD); and wedge-shaped defect (WD). In addition, due to pronounced variability in the bone tunnel models, a sub-classification was provided according to the number of cortex penetrated or whether only the intramedullary cavity was involved: BT.i: bone tunnel along the direction of the intramedullary cavity; BT.u: bone tunnel with uni-cortical penetration; and BT.b: bone tunnel with bi-cortical penetration.

For surgical devices, the authors also used the information mentioned in Figure 2 with the following five categories: IF.IM.N: internal fixation using intramedullary non-locking nails; IF.IM.L: internal fixation with intramedullary locking nails; IF.PS: internal fixation with a plate-screw system; EF: external fixation; and NF (no fixation): for situations when no fixation devices were applied.

**Figure 7:**
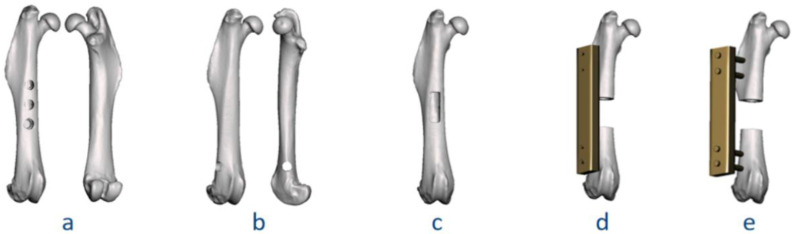
Virtual surgeries for application of the classification system. Coding results: (**a**) II.a-BT.u-NF-(3; 2 mm); (**b**) II.b-BT.b-NF-(1; 2 mm); (**c**) II.a-CW-NF-(1; 6 mm, 2 mm); (**d**) II.a-SD-IF.PS-(1; 6 mm); (**e**) II.a-SD-EF-(1; 6 mm).

To further explain the classification system for application, the authors performed five virtual surgeries involving preparation of the bone tunnels and cortical window, in addition to segmental osteotomy. More details are described in Figure 7 and Table 1. Each surgery was categorized with a classification code, in the following format: [Anatomical location]-[Modeling method]-[Fixation device]-[Defect quantity; Defect size].

### 3.3. Analysis of Potential Surgical Pitfalls

**Figure 8:**
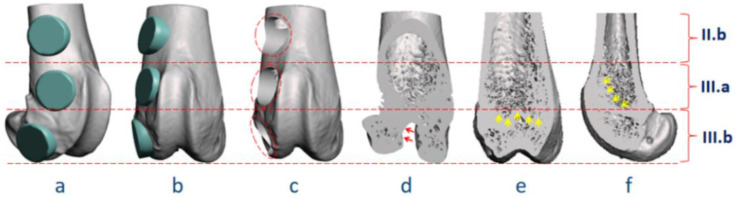
Virtual surgery and anatomical demonstration of potential surgical pitfalls in the distal femur. (**a**–**c**) Virtual surgery simulating the preparation of bone tunnels in anatomical Region II.b, III.a, and III.b; (**d**–**f**) three-dimensional reconstruction of the distal femur, showing the risk of growth plate (indicated by yellow arrows) injury in Region III, and condyle and ligament (indicated by red arrows) injury in Region III.b. Note: (**a**) Oblique-lateral view for surgical design; (**b**) ventral-dorsal view for surgical design; (**c**) ventral-dorsal view after virtual surgery; (**d**) oblique-frontal plane; (**e**) frontal plane; and (**f**) sagittal plane.

For the visualization of complication risks at the distal femur, the authors prepared bone tunnel defects (3 mm in diameter) with virtual surgeries in Region II.b, III.a, and III.b (Figure 8a–c). The following potential pitfalls could be identified through the surgical simulation in combination with normal anatomical images (Figure 8d–f): (1) Transverse bone tunnels in Region III could penetrate the growth plate area (indicated by yellow arrows in Figure 8e,f), a situation close to the physeal injury model, rather than common lesions at the distal femur in human adults [64]; (2) transverse bone tunnels in Region III.b could cause penetration or even fracture in the femoral condyle (red arrows in Figure 8d), and lead to concurrent intra-articular ligament (attachment site also indicated by red arrows) injury, causing persistent joint instability and degeneration [33,65]. In addition, surgical procedures in Region III may result in persistent inflammation and biochemical changes in the local environment of synovial joints, affecting research outcomes related to the degradation and tissue compatibility of biomaterials [66,67]. Region II.b could be a relatively safe area for the preparation of bone tunnels, without the above risks.

### 3.4. Detection of Gender-Related Bone Size Difference

**Figure 9:**
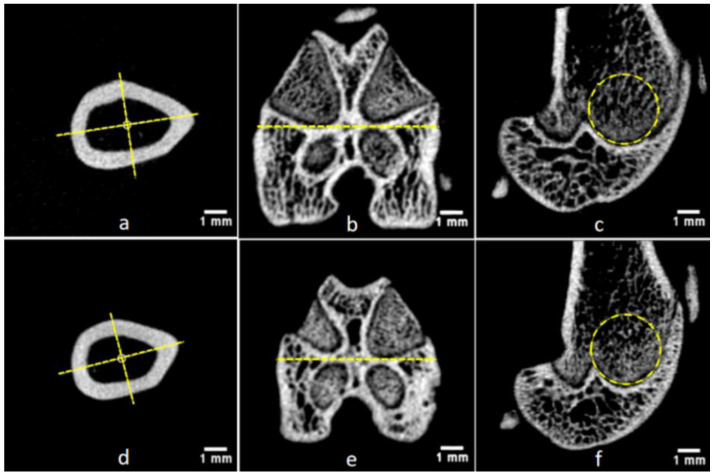
Comparison of bone size between male and female rats. Geometric parameters in the male SD rat (**a**–**c**, age 20 weeks, 556 g) were higher than in the female SD rat (**d**–**f,** age 24 weeks, 338 g). (**a**,**d**) Transverse micro-CT scans of the femoral shaft, at the middle level of the whole femur length: Bone perimeter, male 14.99 mm, female 13.42 mm; minimum caliper width, male 3.70 mm, female 3.33 mm; maximum caliper width, male 5.33 mm, female 4.73 mm. The dashed lines indicate the direction for the measurement of the minimum and maximum caliper width; (**b**,**e**) transverse scans of the distal femur and transverse width of the femoral condyle: male 7.69 mm, female 6.86 mm. The dashed lines indicate the direction of measurement; (**c**,**f**) sagittal scans of the distal femur, with the dashed circles indicating the size of bone tunnels with a diameter of 3 mm.

For female and male SD rats of close age, the body weight and geometric bone parameters would be higher in the male rat as reported in the literature [68,69,70,71,72]. To render the gender difference in bone that might influence the surgical design of femoral defect models, the authors conducted measurements on micro-CT images, at the middle level of the whole femoral length (in Region II.a; Figure 9a,d), and near the boundary level between Region III.a and III.b (Figure 9b,e). The CT images and measurements provided a direct visualization of the relatively wider operating space in the male rat for surgical procedures such as drilling (Figure 9c,f). The gender difference in bone size should be taken into account when selecting surgical instruments and the defect size for a bone tunnel and cortical window to avoid potential complications such as postoperative pathological fractures. Future imaging measurements of bone tissue specifically for rats of different age and gender could further support the precise design of femoral defect surgeries.

## 4. Discussion

Early research using animal models to study bone defect and grafting dated back to the 19th century, and surgical treatment of bone defects in the lower extremities using bone grafts has also been practiced for more than a century [73,74]. However, despite the numerous publications of animal experiments, the status of autograft as the gold standard has remained unchanged [75]. This situation obliges researchers to reflect on how to avoid non-translatable experiments, and to effectively find solutions for challenges in clinical treatment. Evidence-based literature research such as systematic review and meta-analysis can provide objective and comprehensive evaluations for current in vivo studies and identify deficiencies in methodology as good practice of the 3R and 6R principles [76,77].

The fact that surgical techniques have a profound impact on experimental results has long been recognized and is clearly articulated in the ISO standard [14]. However, the guidelines are generally limited to basic surgical preparation such as the aseptic technique and more detailed standardization is needed in real-world studies to minimize the methodological heterogeneity in animal surgeries. Although there were systematic reviews on bone defect models for biomaterials research, the researchers tended to focus on the selection of animal species, evaluation methods, and the comparison of the final results [15,16,78]. To the authors’ knowledge, there were no previous systematic reviews focusing specifically on the surgical techniques of rat femoral defect models. Despite the lack of meta-analysis due to the limitation in data properties, with a systematic review it was still possible to comprehensively analyze the anatomical location, modeling techniques, and fixation devices, thus promoting the standardization of future studies [79,80].

It is noteworthy that more than half of the studies selected relatively simple surgical methods without bone fixation, especially at distal sites (Region II.b and III) [30,31,32,33]. In contrast to human adults, the cartilaginous growth plates remain open in adult rats [81]. When region III is selected for the preparation of bone defects, the pattern of injury and subsequent tissue repair would match more closely to clinical physeal injuries, rather than distal femoral defects in adults [64]. Though these areas are large in size, simple for surgical exposure, and do not require retraction of thick muscle groups, the clinical relevance should be carefully weighed. Such growth plate injuries could be avoided when preparing bone defects in Region II. However, researchers need to be aware that a cylindrical defect at Region II may bring about difficulty in bleeding control or filling with adequate graft materials [34]. The cortical window would be more convenient for hemostasis and material filling. In addition, the cortical window is viewed as a better simulation of skeletal tumor resection than the bone tunnel model [3,82].

When possible, both female and male rats should be included in preclinical studies to avoid the translation being affected. However, there were notable sex differences in terms of growth curves and bone size within the same rat strain [14,70,71,72]. A safe and effective size of a bone tunnel or cortical window in male rats might lead to a high incidence of postoperative pathological fractures in females due to the smaller skeletal dimensions. To the authors’ knowledge, there were no previous studies specifically addressing defect size and postoperative complications for reference, and for ethical reasons, such experiments might be difficult to perform. Comprehensive and objective reporting of postoperative complications, in combination with preoperative surgical simulation or finite element analysis based on imaging data, could provide beneficial support during experimental planning.

In addition, gender differences were reported in the capacity for bone regeneration, which could be more rapid in male rats than in females [83]. This should be taken into account when setting the follow-up period for female rats with reference from previous studies in males. A longer follow-up period might be reasonable for female rats with reduced bone repair capacity such as osteoporosis, and similar situations also exist for animal studies with diabetes or other comorbidities [84,85].

There were variations in the gap size of segmental (2−10 mm) and wedge-shaped defect (3−5 mm) models [37,38,39,45,46,47]. Although the application of fixation devices ensured bone stabilization, the defect size and follow-up setting should be taken into consideration when comparing results from different studies. However, given the variability in defect sizes and animal status, there was also no specific consensus on the setting of follow-up for reference. Based on the information from the included studies, the authors recommend the selection of a critical gap size larger than 4 mm [43,44], a maximum follow-up of no less than 6 weeks for bone tunnel or cortical window models, and no less than 8−12 weeks for the osteotomy defects [29,30,31,32,34,35,36,38,39,40]. The impact of gender and defect size on the setting of follow-up periods might be further addressed in future animal studies.

The fixation device was also a main source of heterogeneity in animal studies. The surgical instrumentation for rodent femoral defect studies has been optimized in recent years, allowing effective simulation of clinical treatment in humans. However, intramedullary nailing with a locking mechanism has not been widely used in published studies [40,48,49,50,51]. Some plating fixation involved only metal wires instead of screws to provide stability [57,58], and external fixators with higher technical difficulty were selected less often than internal fixation [86]. The application of fine instruments that highly mimic clinical treatments might also be limited by the potential high price of standardized devices. As 3D printing devices become more available, fixation devices that can be prepared by rapid manufacturing may contribute significantly to the standardization of preclinical animal models. Moreover, the application of non-locking intramedullary pins without rotational stability should be avoided, as this is no longer a standard technique for adult lower extremity fractures [87,88,89].

**Figure 10:**
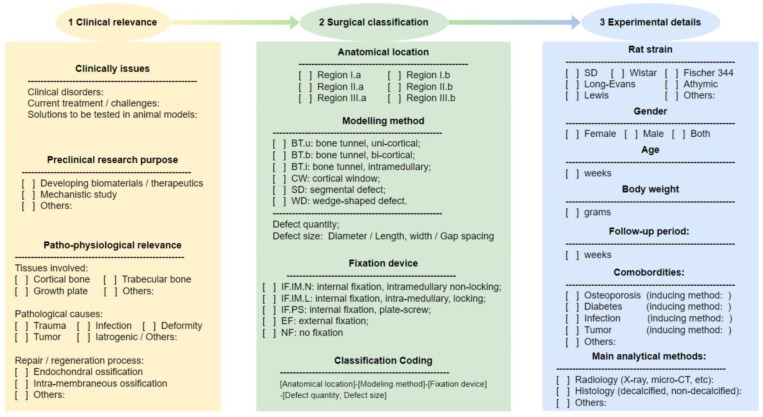
Application pipeline involving the surgical classification system for experimental planning.

Regardless of the research purpose or surgical strategy, explicit clinical relevance is the fundamental prerequisite before conducting preclinical animal studies. Although routine descriptions were generally available in publications, however, most were overly generalized statements about clinical problems without an effective connection between surgical methods and specific patho-physiological mechanisms [90,91]. Moreover, due to the various purposes of the included research, the reporting of surgical details was relatively limited and fragmented in different sections of papers, making it difficult to extract related information for an evidence-based analysis. In order to improve the limited translational prospects and to better implement the surgical classification system, the authors established an application workflow incorporating the classification system and surgery-related details for experimental planning, in particular regarding the clinical relevance (Figure 10).

## 5. Conclusions

This systematic review in combination with 3D analysis and virtual surgery provided a detailed overview of current surgical approaches for inducing rat femoral defects, and established a classification system to facilitate future preclinical research with improved quality and translational value.

## Figures and Tables

**Table 1:** Detailed explanation of the classification coding for virtual surgeries in Figure 7.

Figure	Classification Code	Detailed Explanation			
		Anatomical Region	Surgical Method	Fixation Device	Defect Quantity; Defect Size
7a	II.a-BT.u-NF-(3; 2 mm)	II.a	BT.u: uni-cortical tunnel	NF: no fixation	Quantity: 3; Diameter: 2 mm
7b	II.b-BT.b-NF-(1; 2 mm)	II.b	BT.b: bi-cortical tunnel	NF: no fixation	Quantity: 1; Diameter: 2 mm
7c	II.a-CW-NF-(1; 6 mm, 2 mm)	II.a	CW: cortical window	NF: no fixation	Quantity: 1; Length: 6 mm, width: 2 mm
7d	II.a-SD-IF.PS-(1; 6 mm)	II.a	SD: segmental defect	IF.PS: internal fixation, plate-screw	Quantity: 1; Gap size: 6 mm
7e	II.a-SD-EF-(1; 6 mm)	II.a	SD: segmental defect	EF: external fixation	Quantity: 1; Gap size: 6 mm

## Data Availability

The data that support the findings of this study are available from the corresponding author upon reasonable request.

## Supplementary Materials

The following supporting information can be downloaded at: www.mdpi.com/article/10.3390/bioengineering9090476/s1, Table S1. PubMed search list.
